# TOPAZ: asymmetric suffix array neighbourhood search for massive protein databases

**DOI:** 10.1186/s12859-018-2290-3

**Published:** 2018-07-31

**Authors:** Alan Medlar, Liisa Holm

**Affiliations:** Institute of Biotechnology, University of Helsinki, Helsinki, 00014 Finland

**Keywords:** Homology search, Suffix arrays, BLAST

## Abstract

**Background:**

Protein homology search is an important, yet time-consuming, step in everything from protein annotation to metagenomics. Its application, however, has become increasingly challenging, due to the exponential growth of protein databases. In order to perform homology search at the required scale, many methods have been proposed as alternatives to BLAST that make an explicit trade-off between sensitivity and speed. One such method, SANSparallel, uses a parallel implementation of the suffix array neighbourhood search (SANS) technique to achieve high speed and provides several modes to allow for greater sensitivity at the expense of performance.

**Results:**

We present a new approach called asymmetric SANS together with scored seeds and an alternative suffix array ordering scheme called optimal substitution ordering. These techniques dramatically improve both the sensitivity and speed of the SANS approach. Our implementation, TOPAZ, is one of the top performing methods in terms of speed, sensitivity and scalability. In our benchmark, searching UniProtKB for homologous proteins to the *Dickeya solani* proteome, TOPAZ took less than 3 minutes to achieve a sensitivity of 0.84 compared to BLAST.

**Conclusions:**

Despite the trade-off homology search methods have to make between sensitivity and speed, TOPAZ stands out as one of the most sensitive and highest performance methods currently available.

**Electronic supplementary material:**

The online version of this article (10.1186/s12859-018-2290-3) contains supplementary material, which is available to authorized users.

## Background

Protein homology search is the most common analysis task performed in bioinformatics. Unfortunately, the exponential growth of protein databases and the rising demands of high-throughput experiments are creating a computational bottleneck for what was previously a routine task. This is a problem because homology search is a crucial step in many data-intensive applications, such as functional annotation [1], metagenomics [2], comparative genomics [3] and evolutionary analysis [4]. In addition to high-throughput experiments, time-sensitive applications in clinical settings are dependent on the performance of homology search. For example, with sequence-based diagnostics for identifying bacterial infections, including pathogen outbreaks and antibiotic resistance [5], a late diagnosis could result in death.

The gold standard for homology search is BLAST [6]. BLAST uses a seed-and-extend approach to perform database search. In brief, BLAST uses heuristics based on amino acid substitution rates to identify initial matches, or seeds, between query and database sequences. These matches are then extended into local alignments to avoid the computational overhead of full dynamic programming. While BLAST is highly sensitive, its runtime scales linearly with the size of the database. BLAST’s performance can be improved with parallelism, but further speedups are only possible at the expense of sensitivity.

With this trade-off in mind, there are numerous BLAST alternatives for fast homology search. Many of the fastest methods use either an uncompressed suffix array [7] or FM-index [8], a compressed full-text index based on the Burrows-Wheeler transform [9]. SANSparallel, for example, uses the concept of a suffix array neighbourhood (described in methods) to identify proteins which would be more frequently co-located in the suffix array with the query sequence. These proteins are ranked and the top hits aligned [10, 11]. LAST uses an uncompressed suffix array to find adaptive seeds, which are initial sequence matches that are variable length and defined by their multiplicity [12]. LAST additionally uses a reduced amino acid alphabet to improve sensitivity [13]. Lambda uses a reduced alphabet, double indexing (indexing seeds from both query and database sequences) and multiple backtracking of fixed length seeds to achieve high speed [14]. Finally, DIAMOND uses a reduced alphabet, double indexing and spaced seeds [15] to achieve higher sensitivity [16]. While these methods all use similar techniques, their performance differs considerably.

In this article we present TOPAZ, a fast and sensitive homology search method. TOPAZ is based on an extension of the suffix array neighbourhood search (SANS) concept used by SANSparallel, called asymmetric SANS. Asymmetric SANS uses scored seeds and a suffix array ordering called optimal substitution ordering to improve the speed and sensitivity of SANS. In our evaluation, we focus on three metrics: speed, sensitivity and scalability. TOPAZ is one of the best performing methods for each evaluation metric, despite the inherent trade-offs involved.

## Implementation

Protein homology search methods tend to follow the same basic template. Protein sequences are held in a database that is queried with a set of query sequences using the following procedure for each query:

1. Find initial sequence matches (seeds)
2. Perform local alignment on a subset of those matches
3. Output the top hits meeting some user-defined criteria

These user-defined criteria include variables such as statistical significance and maximum number of hits per query. We will first describe how suffix array neighbourhood search (SANS) carries out this procedure, then the components of asymmetric SANS and how it is implemented in TOPAZ.

### Suffix array neighbourhood search (SANS)

The SANS method uses an uncompressed suffix array to hold a set of proteins, *P*. A suffix array, *SA*, is defined as an array *SA*[1..*n*] in which *SA*[*j*]=*i* iff *T*[*i*..*n*] is the *j*th suffix of *T* in lexicographical order. In our case, *T* is the concatenation of the set of proteins, *P*, separated by a delimiter character.

**Fig. 1:**
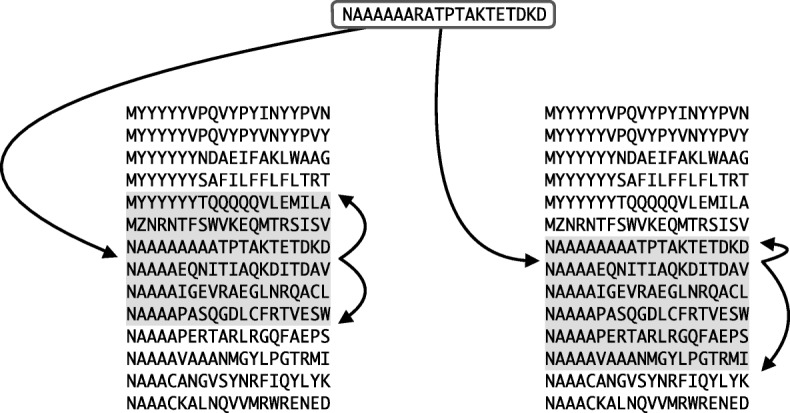
Suffix array windows. Left: Suffix array neighbourhood search, the insertion point of the query suffix is found and the proteins containing the suffixes from a symmetric window in the suffix array receive votes. Right: Asymmetric suffix array neighbourhood search, the window is not necessarily symmetric, but extends greedily based on the ungapped alignment score between query and database suffixes

Each query sequence, *Q*, is split into suffixes, *Q*[*i*..*n*], and *k* is the position in the suffix array where *Q*[*i*..*n*] would be inserted. As *SA* is in lexicographical order, the position of *Q*[*i*..*n*] can be found in *O*(log|*T*|) time using binary search. Proteins in the database accumulate votes if they contain a suffix that falls into a fixed-length window, *W*, surrounding position *k* (see Fig. 1, left). For each suffix contained in *W*, the originating protein gets 1 vote. The top *N* proteins in descending order of vote count are aligned and, of these, the top *H* proteins by alignment score are output.

### Asymmetric SANS

SANS is highly efficient, but can be suboptimal in boundary cases where the position *k* is directly before or after a contiguous block of database suffixes that have low identity to the query suffix (Fig. 1). More generally, if we consider that we have a static number of votes, *V*, where *V*=|*Q*|·*W*, then we do not necessarily want to treat each suffix equally as SANS does. Ideally, we want to weight the importance of each query suffix by the degree of similarity with the surrounding suffixes in *SA*.

Figure 1 (right) shows how an asymmetric window would work, using the boundary case as an example. The window originally centred around position *k* is now defined by *k*_*upper*_ and *k*_*lower*_ that are greedily expanded based on the sequence similarity between the query suffix and the database suffix at the edge of the window. Asymmetric SANS applies the total number of votes, *V*, across all suffix windows, allowing it to focus on the most “promising” areas of the suffix array. Indeed, some suffixes may not contribute to the final result at all if they are only surrounded by dissimilar sequences.

Algorithm 1 describes the asymmetric SANS algorithm. For priority queues, we use red-black trees because they are self-balancing, making the worst-case lookup time *O*(log*n*) [17]. The functions *push*(), *pop*_*lowest*() and *pop*_*highest*() are functions that push items on to the queue, pop the item with the lowest and highest priority. *align*() performs a pair-wise local alignment using a substitution matrix specified by the user. *increment*() increments the position if it is an upper bound or decrements the position if it is a lower bound of a window. *get*_*protein*() retrieves the protein associated with the suffix at a given position in the suffix array.


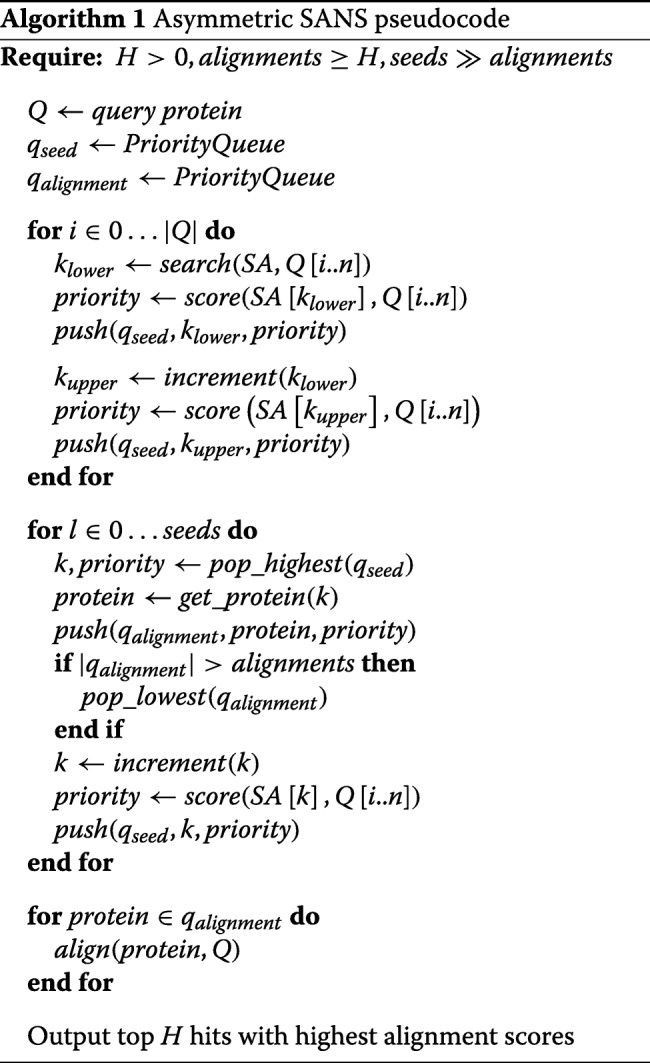


### Scored seeds

In Algorithm 1 we did not define the function *score*(), which is used to greedily increase the extents of the windows in the suffix array. The frontier of each window is given a score equal to the maximum gapless alignment score between query suffix and the suffix found at the current position in the suffix array:

1 $$ \underset{n}{\mathrm{arg\,max}} \sum\limits_{i}^{n} M(Q\left[i\right], T\left[SA\left[k\right]+i\right])  $$

where *Q* is the query suffix, *n*=1..|*Q*|, *k* is the current position in the suffix array, *SA*, and *M* is an amino acid substitution matrix. In the current implementation, the same substitution matrix is used for scoring and alignment. Sequences are repeat masked with SEG [18] during scoring.

We note that using a gapless alignment score in this manner is similar to a spaced seed, where a bitmask of 1s and 0s defines match and “don’t-care” positions, respectively [15]. By maximising the gapless alignment score, we are effectively using a spaced seed that is variable length and the bit pattern is not defined a priori. We refer to these as *scored seeds*.

### Optimal substitution ordering

Suffix arrays are usually sorted into lexicographical order. However, for protein sequences this is clearly suboptimal, for example, Cysteine (C) and Aspartic acid (D) are lexicographically consecutive, but have a substitution score of -3 in BLOSUM62.

In order to find the optimal ordering of amino acids, i.e. the ordering that minimises the summation of substitution scores between consecutive letters (and between the first and last letter), we cast the problem as the traveling salesman problem (TSP). Instead of cities we have amino acids and instead of distances between cities we have substitution scores. We used substitution scores from BLOSUM62 and converted them to quasi-distances by negating the score and adding 5. Distances between an amino acid and itself were set to 0.

We used the Concorde TSP solver (http://www.math.uwaterloo.ca/tsp/concorde/) on the NEOS server [19] to find the optimal substitution ordering of amino acids. The optimal solution was found to be: ACMLJIVTSKRQZEDBNHYFWXP*G however, we note that there are many equally good solutions.

### TOPAZ implementation

We provide an implementation of asymmetric SANS called TOPAZ. TOPAZ is written in C and uses libdivsufsort (https://github.com/y-256/libdivsufsort) for suffix array construction and the SSW library for local alignment [20].

## Results

We compare the performance of TOPAZ with BLAST (ver. 2.5.0+) [6], DIAMOND (ver. 0.8.37.99) [16], Lambda (ver. 1.9.2) [14], LAST (ver. 801) [12] and SANSparallel (ver. 2.2) [11]. While there are many other methods for protein homology search, we focused on methods that have demonstrated good performance in previous benchmarks (see [11]).

### Experimental setup

#### Data sets

We used the complete UniProtKB database (downloaded March 2017) containing 78 million protein sequences. For query sequences we used the *Dickeya solani* proteome (4174 sequences), unless otherwise stated. While these sequences are themselves contained in UniProtKB, they contain a mixture of “easy” queries, where there are many similar sequences in the database and “harder” queries where BLAST finds very few significant hits.

#### Program options

Where possible, each method was run to output 1000 hits per query sequence with an E-value less than or equal to 1. As some methods output more than 1000 search results per query, we only kept the top 1000 hits by bitscore. Each program was run using 1, 2, 4, 8, 16, 32 and 64 threads to assess scalability. Timing measurements were taken by running the program twice and using the measurement from the second run to ensure disk access times were not a factor. These parameter values were chosen to emphasise the importance of sensitivity, however, we additionally ran all methods with an E-value threshold 10^−9^, outputting 100 and 1000 hits (see Additional file 1). For BLAST, DIAMOND and TOPAZ these parameter differences do not affect the runtime. We note, however, that reducing the maxmimum number of hits increased the speed of Lambda and SANSparallel, and a more stringent E-value threshold increased the runtime of LAST.

To make this a fair test, we additionally ran each method in different modes to trade-off speed and sensitivity. While we have attempted to fairly represent the performance of each method, we make no claim that these are the best results possible with each program. SANSparallel has several protocols: verifast, fast, slow and verislow. The verifast mode does not calculate E-values and was therefore omitted. We ran Lambda for faster, lower sensitivity protein searches (using options -so 5 -sh on) and slower, higher sensitivity (-so 5). While we additionally ran Lambda with default options, it was both slower and less sensitive than fast mode, so the results were omitted. The Lambda database was constructed using the Murphy10 alphabet and an FM-index. DIAMOND was run with default parameters, in sensitive mode (--sensitive) and more sensitive mode (--more-sensitive). For LAST, the maximum number of hits to output cannot be specified. It does, however, allow us to specify the maximum number of initial matches per query suffix (using option -m). After some experimentation, we decided to run *m*= 100, 1000 and 10,000 as these values gave similar sensitivity results to other methods. TOPAZ was run with default parameters (--seeds 300000 --alignments 5000) and with alternate parameters to emphasise speed over sensitivity (--seeds 100000 --alignments 1500). BLAST was run with default parameters. The results presented in Table 1 show the overall sensitivity, runtimes using different numbers of threads and the peak memory usage of each method.

**Table 1:** Runtimes using different numbers of threads and overall sensitivity compared to BLAST results for all methods tested

		Runtime using N threads (seconds)							Mem.
Method	Sens.	1	2	4	8	16	32	64	(GB)
BLAST	1.0	1,801,293	948,124	479,400	247,715	128,401	70,107	43,647	9.9
DIAMOND (default)	0.840	41,863	21,330	10,677	5520	3269	2259	1919	8.6
DIAMOND (sensitive)	0.926	137,100	67,858	35,615	19,077	10,452	8463	7723	10.0
DIAMOND (more sens.)	0.931	166,737	85,689	45,954	23,716	12,849	11,204	10,174	12.0
Lambda (fast)	0.681	3325	1916	1152	776	586	443	435	104.2
Lambda (sensitive)	0.726	5200	2819	1636	940	631	477	456	104.3
LAST (*m*=100)	0.636	**1714**	**946**	**525**	326	233	166	141	20.9
LAST (*m*=1000)	0.838	7993	4397	2308	1381	859	585	439	24.3
LAST (*m*=10000)	0.881	33,411	17,859	9473	5224	3096	1891	1347	30.1
SANSparallel (fast)	0.696	6301	2938	1573	992	577	433	472	230.4
SANSparallel (slow)	0.758	11,258	5123	2801	1770	1150	985	1247	230.4
SANSparallel (verislow)	0.779	18,039	8396	4330	2639	1639	1305	1551	230.4
TOPAZ (fast)	0.800	2267	1111	569	**299**	**160**	**99**	**78**	57.2
TOPAZ (default)	0.840	5961	2961	1559	779	418	243	175	60.5

TOPAZ (default) has similar sensitivity to LAST (*m*=1000) and DIAMOND (default), but is faster than both methods irrespective of the number of threads. Bold indicates the fastest method for each number of threads. TOPAZ (fast) is the fastest method for 8–64 threads. LAST (*m*=100) is the fastest method for 1–4 threads, but suffers from the lowest sensitivity

### Sensitivity

**Fig. 2:**
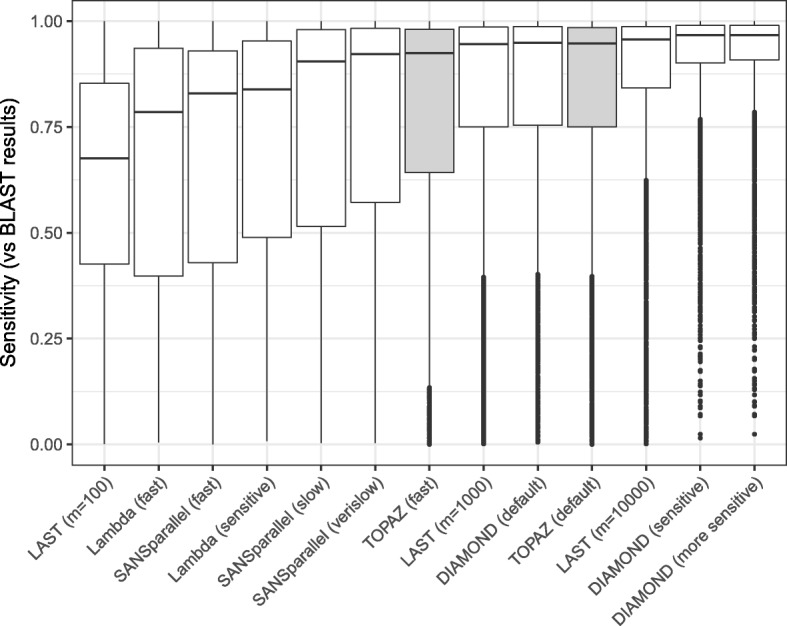
Distribution of sensitivity values per protein compared with BLAST results for each method. Methods are ordered by mean sensitivity. TOPAZ modes are highlighted in grey

Figure 2 shows boxplots of sensitivity values for each protein in the query set ordered by mean sensitivity. As we did not have the ground truth for the entire data set, we instead calculated the sensitivity of each method by comparing with BLAST results. For each query, we removed BLAST results with bitscores equal to the bitscore of the 1000^*th*^ hit, if it exists (i.e. if there are at least 1000 hits). This removes the potential for rank ambiguity if, for example, a search method were to return what would be the 1001^*st*^ BLAST result with the same bitscore as the 1000^*th*^ result. This procedure resulted in the removal of 0.9% of BLAST results.

The results show a wide range of sensitivity values for all methods. The faster run modes (LAST (*m*=100), Lambda (fast), SANSparallel (fast)) have the lowest average sensitivity. TOPAZ (default) has the 4^*th*^ highest average sensitivity, with only LAST (*m*=10,000) and both of DIAMOND’s non-default modes being higher.

With more stringent E-value thresholds, while the ranking stayed broadly the same, the gap in average sensitivity narrowed (see Additional file 1: Figures S1 and S3). For example, the average sensitivity for DIAMOND (more sensitive) was 0.11 higher than TOPAZ (default) with E-value threshold 1, but decreased to 0.07 with an E-value threshold of 10^−9^. When outputting only 100 hits with an E-value threshold of 10^−9^, the difference further decreased to 0.03.

### Speed/sensitivity trade-off

**Fig. 3:**
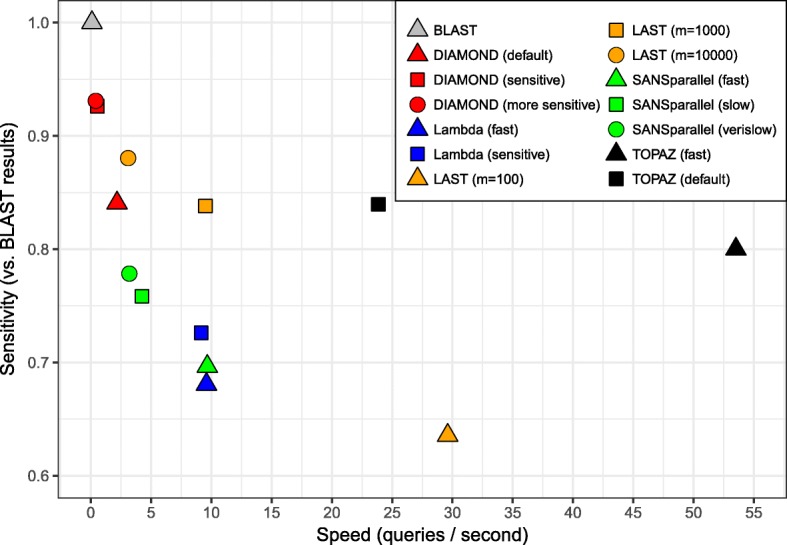
Speed versus average sensitivity across all proteins. The best speed was used for each method using up to 64 threads (all methods used 64 threads, with the exception of SANSparallel, which used 32)

While sensitivity is important, all methods make a trade-off between sensitivity and speed. We show this trade-off in Fig. 3. Sensitivity was calculated over all search queries, again using the BLAST results as the ground truth. Runtime was the fastest time using any number of threads (see Table 1). For all methods, the fastest runtime was obtained with 64 threads, with the exception of SANSparallel (all run modes), where 32 threads was fastest (this was likely due to communication overhead in MPI). The perfect method would be in the top-right corner of the figure, with perfect sensitivity and high speed.

As Fig. 3 shows, faster methods tend to be less sensitive. However, TOPAZ has high speed while sacrificing less sensitivity. The only method faster than TOPAZ (default) is LAST (*m*=100) which has the lowest sensitivity of all methods (Fig. 2). TOPAZ (fast) is the fastest method overall, while being more sensitive than SANSparallel and Lambda (all modes).

The four methods with higher sensitivity than TOPAZ (default) (LAST (*m*=10000), DIAMOND (sensitive), DIAMOND (more sensitive) and BLAST) have far longer runtimes: 7.7 ×, 44.1 ×, 58.1 × and 249.4 ×, respectively. Even methods with similar sensitivity had longer runtimes: LAST (*m*=1000) took 2.5 × longer and DIAMOND (default) took 11.0 × longer to run. The same trend is observed at more stringent E-value thresholds (Additional file 1: Figure S2) and for fewer hits (Additional file 1: Figure S4).

### Parallel scalability

**Fig. 4:**
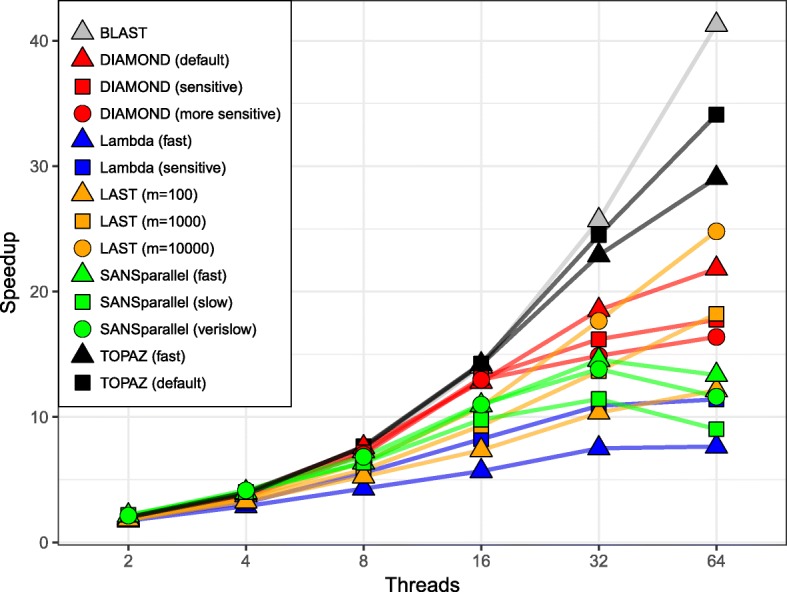
Speedup versus the number of threads. Speedup is defined as the runtime using 1 thread divided by the runtime with *n* threads. For 16–64 threads TOPAZ and BLAST achieved the highest speedup

Figure 4 shows the speedup using different numbers of threads concurrently. Speedup is *r*_1_/*r*_*n*_, where *n* is the number of threads and *r*_*n*_ is the runtime using *n* threads. With zero overhead, the speedup would be equal to the number of threads.

At higher numbers of threads (16-64), BLAST was consistently the most efficient, followed by TOPAZ. For example, at 64 threads BLAST and TOPAZ had speedups of 41.3 × and 34.1 ×, respectively. BLAST, however, is doing much more work per query and, therefore, has less communication overhead allowing it to be highly parallel. At lower numbers of threads (2–4), both DIAMOND (all modes) and SANSparallel (all modes) had the highest efficiency.

### Input size scalability

**Fig. 5:**
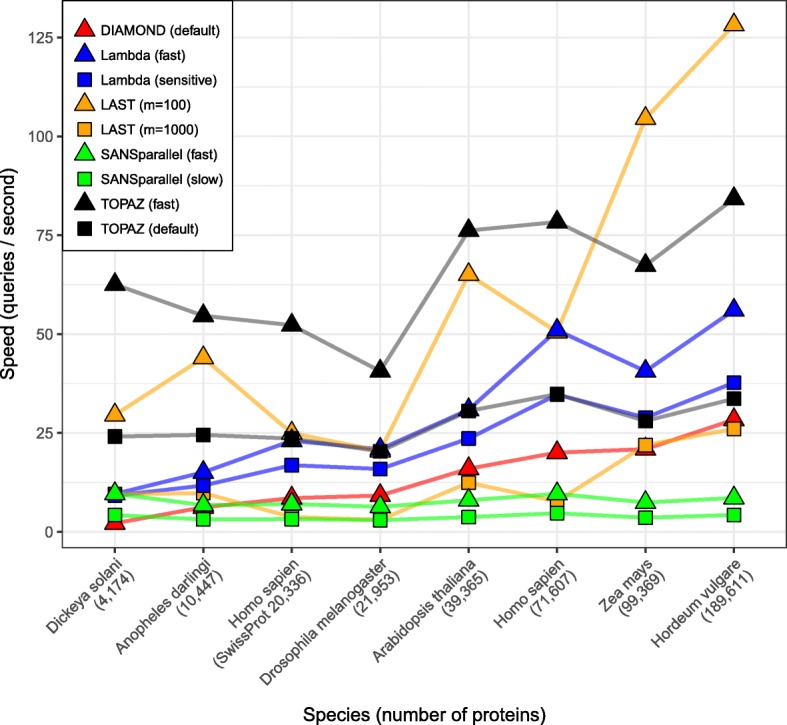
Speed in queries per second for the fastest homology search methods. Query sets were 8 different proteomes containing 4,174–189,611 query sequences. TOPAZ (fast) is the fastest method in 6/8 proteomes

To understand how each method scales with query set size, we tested the fastest methods on increasingly large proteomes. We used the following proteomes as query sets: *Dickeya solani* (4174 sequences), *Anopheles darlingi* (10,447), *Homo sapien* (SwissProt only, 20,336), *Drosophila melanogaster* (21,953), *Arabidopsis thaliana* (39,365), *Homo sapien* (71,607), *Zea mays* (99,369) and *Hordeum vulgare* (189,611). We ran all methods with the exception of BLAST and the most sensitive modes for DIAMOND and SANSparallel due to long runtimes. We did not run LAST (*m*=10000) due to the size of the output files. For Lambda we needed to remove the longest queries from the *Homo sapiens* proteome as these sequences caused the program to crash. We ran all methods with an E-value threshold of 1 and to output a maximum of 1000 hits. All methods were run with 64 threads, with the exception of SANSparallel which was run with 32. The results are shown in Fig. 5.

For 6 of the 8 proteomes, TOPAZ (fast) was the fastest method. The second fastest method, LAST (*m*=100), was previously shown to be the least sensitive for these parameter settings. In general, the fastest methods tended to be those shown previously as having lower sensitivity (Lambda (both modes) and LAST (*m*=100)), with the exception of TOPAZ (both modes). We had expected DIAMOND to be faster as the cost of online indexing should be amortised over large query sets, but it appears to scale similarly to methods that process queries individually. It is possible that this efficiency is only realised with query sets larger than the *H. vulgare* proteome. Unlike other methods, SANSparallel has constant speed, irrespective of query set. This is detrimental in lesser studied organisms where there are simply fewer significant alignments to be found.

### Optimal substitution versus lexicographical ordering

Using optimal substitution ordering for building the suffix array in TOPAZ (default) resulted in higher sensitivities for 1395/4174 *Dickeya solani* proteins (average difference = 21.8 extra hits per protein) and lower sensitivities for 548 proteins (average difference = 2.0 less hits per protein) compared with lexicographical ordering. Across all proteins, optimal substitution ordering gave 7.1 more hits per protein on average than lexicographical ordering. While we acknowledge this is a modest improvement, as we are simply redefining the ordering of amino acids, there is no performance penalty.

## Discussion and conclusions

We presented TOPAZ, a protein homology search method based on asymmetric suffix array neighbourhood search, scored seeds and optimal substitution ordering. All BLAST alternatives trade-off sensitivity in exchange for speed. In doing so, database search can be used in high-throughput and time-sensitive applications that would have otherwise taken a prohibitively long time. This trade-off was considered at all points in TOPAZ’s development, where our design goals were speed, sensitivity and the efficient use of parallelism.

We have demonstrated that TOPAZ is one of the most sensitive and fastest homology search methods. TOPAZ had one of the highest average sensitivity scores (Fig. 2), whereas more sensitive methods had 8–250 × longer runtimes (Fig. 3). Similarly, the only method that was faster than TOPAZ had the worst average sensitivity (Fig. 2). TOPAZ’s speed comes from how efficiently it uses the processing power available to it (Fig. 4). TOPAZ was the second most efficient method using 16–64 threads with only BLAST scaling better. Across a range of query set sizes TOPAZ (fast) was the fastest method in a majority of cases and TOPAZ (default) was consistently faster than methods which had previously shown similar sensitivity (Fig. 5).

The fastest methods tended to have the highest peak memory usages (Table 1). From one perspective high memory usage is not a problem because servers are increasingly well provisioned for data-intensive applications. However, the exponential growth of protein databases suggests that this might become a problem in the future. TOPAZ makes extensive use of memory-mapped IO to ensure that the operating system can move parts of the database in and out of memory as the workload changes. Other techniques could be used to mitigate this issue, for example, LAST builds multiple suffix arrays using 32 bit integers. While this limits the maximum size of the database to 4GB, it is overcome by splitting the database into multiple partitions. Despite the added complexity of moving from 64 to 32 bits, it has the added benefit of halving total memory requirements.

While all methods in this study make use of process-level, and possibly instruction-level, parallelism, none make use of alternative architectures such as general purpose GPUs that are increasingly common in computer clusters and desktop computers. While GPU-enabled versions of, for example, BLAST exist [21], the speedups are underwhelming compared with those achieved in other areas of bioinformatics (e.g. [22]). We note, however, that homology search is more data-intensive than applications which have achieved massive performance improvements, making memory size and bandwidth the main impediments to adoption.

Finally, in studies such as this, there is a focus on comparing results with BLAST, which is widely considered the gold standard for homology search. However, to our knowledge, there is no analysis of the downstream effects of different sensitivity scores in different application domains. For example, transfer of functional annotation is only performed at higher similarities and, therefore, does not require highly sensitive search results. We would like to see more analysis on requirements for different domains, enabling research in homology search to have a more application-specific focus.

## Availability and requirements

**Project name:** TOPAZ

**Project home page:** https://github.com/ajm/topaz

**Operating system(s):** Linux

**Programming language:** ANSI C

**Other requirements:** TCMalloc

**License:** GNU GPL version 3

**Any restrictions to use by non-academics:** none

## Additional file

Additional file 1 Supplementary results showing method performance with different parameter settings. (PDF 120 kb)

## Abbreviations

SANS: Suffix array neighbourhood search

## References

[1] Törönen P, Medlar A, Holm L (2018). PANNZER2: a rapid functional annotation web server. Nucleic Acids Res.

[2] Medlar A, Aivelo T, Löytynoja A (2014). Séance: Reference-based phylogenetic analysis for 18s rRNA studies. BMC Evol Biol.

[3] Medlar A, Törönen P, Holm L (2018). AAI-profiler: fast proteome-wide exploratory analysis reveals taxonomic identity, misclassification and contamination. Nucleic Acids Res.

[4] Veidenberg A, Medlar A, Löytynoja A (2015). Wasabi: An integrated platform for evolutionary sequence analysis and data visualization. Mol Biol Evol.

[5] Fournier P-E, Dubourg G, Raoult D (2014). Clinical detection and characterization of bacterial pathogens in the genomics era. Genome Med.

[6] Camacho C, Coulouris G, Avagyan V, Ma N, Papadopoulos J, Bealer K, Madden TL (2009). BLAST+: architecture and applications. BMC Bioinformatics.

[7] Manber U, Myers G (1993). Suffix arrays: A new method for on-line string searches. SIAM J Comput.

[8] Ferragina P, Manzini G (2000). Opportunistic data structures with applications. Foundations of Computer Science, 2000. Proceedings. 41st Annual Symposium On.

[9] Burrows M, Wheeler DJ. A block-sorting lossless data compression algorithm. 1994. Technical report 124, 1994, Digital Equipment Corporation, Palo Alto, CA.

[10] Koskinen JP, Holm L (2012). SANS: High-throughput retrieval of protein sequences allowing 50% mismatches. Bioinformatics.

[11] Somervuo P, Holm L (2015). SANSparallel: Interactive homology search against Uniprot. Nucleic Acids Res.

[12] Kiełbasa SM, Wan R, Sato K, Horton P, Frith MC (2011). Adaptive seeds tame genomic sequence comparison. Genome Res.

[13] Murphy LR, Wallqvist A, Levy RM (2000). Simplified amino acid alphabets for protein fold recognition and implications for folding. Protein Eng.

[14] Hauswedell H, Singer J, Reinert K (2014). Lambda: The local aligner for massive biological data. Bioinformatics.

[15] Ma B, Tromp J, Li M (2002). PatternHunter: faster and more sensitive homology search. Bioinformatics.

[16] Buchfink B, Xie C, Huson DH (2015). Fast and sensitive protein alignment using DIAMOND. Nat Methods.

[17] Cormen TH, Leiserson CE, Rivest RL, Stein C (2009). Introduction to Algorithms.

[18] Wootton JC, Federhen S (1996). Analysis of compositionally biased regions in sequence databases. Methods Enzymol.

[19] Czyzyk J, Mesnier MP, Moré JJ (1998). The NEOS server. IEEE Comput Sci Eng.

[20] Zhao M, Lee W-P, Garrison EP, Marth GT (2013). SSW library: An SIMD Smith-Waterman C/C++ library for use in genomic applications. PloS ONE.

[21] Vouzis PD, Sahinidis NV (2010). GPU-BLAST: Using graphics processors to accelerate protein sequence alignment. Bioinformatics.

[22] Medlar A, Głowacka D, Stanescu H, Bryson K, Kleta R (2012). SwiftLink: Parallel MCMC linkage analysis using multicore CPU and GPU. Bioinformatics.

