# Supplementary material for: TOPAZ: asymmetric suffix array neighbourhood search for massive protein databases
Source: BMC Bioinformatics. 2018 Jul 31;19:278. doi: 10.1186/s12859-018-2290-3 (PMC6069885; doi:10.1186/s12859-018-2290-3)
Supplement: Supplementary file 1 — Supplementary results showing method performance with different parameter settings. (PDF 120 kb) [file 12859_2018_2290_MOESM1_ESM.pdf]

TOPAZ: Asymmetric suffix array neighbourhood search for massive protein databases
Supplementary results

Alan Medlar and Liisa Holm

These supplementary results demonstrate that the sensitivity and speed trends between homology search methods highlighted in the manuscript are predominantly insensitive to the number of hits and E-value threshold (i.e. rankings are highly similar). We used the complete UniProtKB database (downloaded March 2017) containing 78 million protein sequences. For query sequences we used the complete Dickeya solani proteome (4174 sequences). Parameter settings and post-processing of results is the same as the experiments in the main text, with a few exceptions. In Figures S1 and S2 each method output 1000 hits with an E-value threshold 10^{-9}*. In Figures S3 and S4 each method output 100 hits with E-value threshold* 10^{-9}*.*

There are several instances where the trends seen here differ from the main text. More stringent E-value thresholds result in longer runtimes for LAST (all modes). Lower numbers of hits result in shorter runtimes for SANSparallel (all modes) and Lambda (all modes). For BLAST, DIAMOND and TOPAZ, runtimes were similar across all parameters settings.

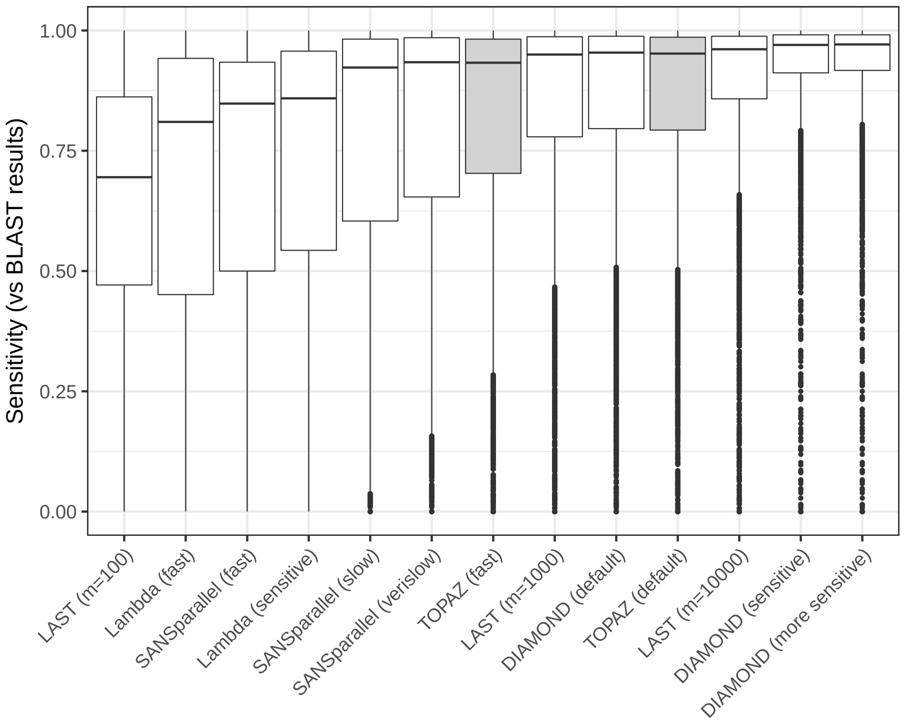

Figure S1: Distribution of sensitivity values per protein compared with BLAST results for each method. Methods are ordered by mean sensitivity. TOPAZ modes are highlighted in grey. Number of hits = 1000 and E-value threshold = 10^{-9}.

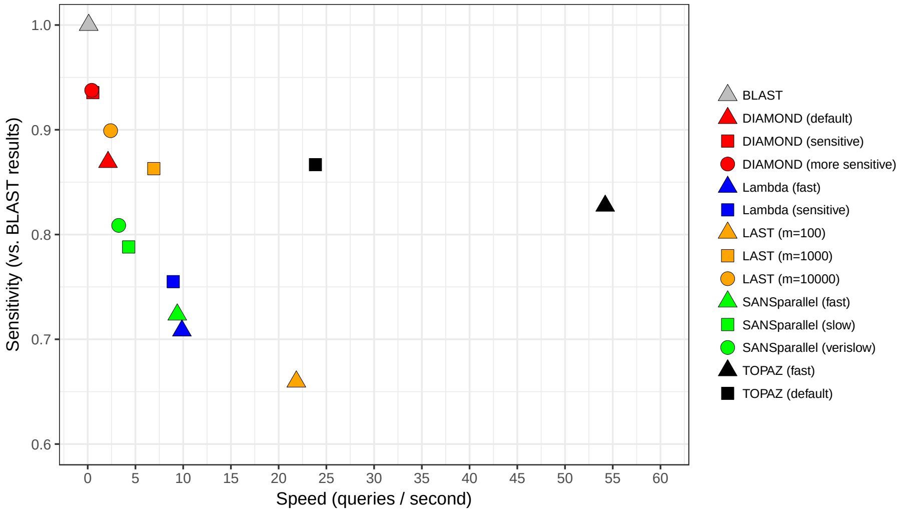

Figure S2: Speed versus average sensitivity across all proteins. The best speed was used for each method using up to 64 threads (all methods used 64 threads, with the exception of SANSparallel, which used 32). Number of hits = 1000 and E-value threshold = 10^{-9}.

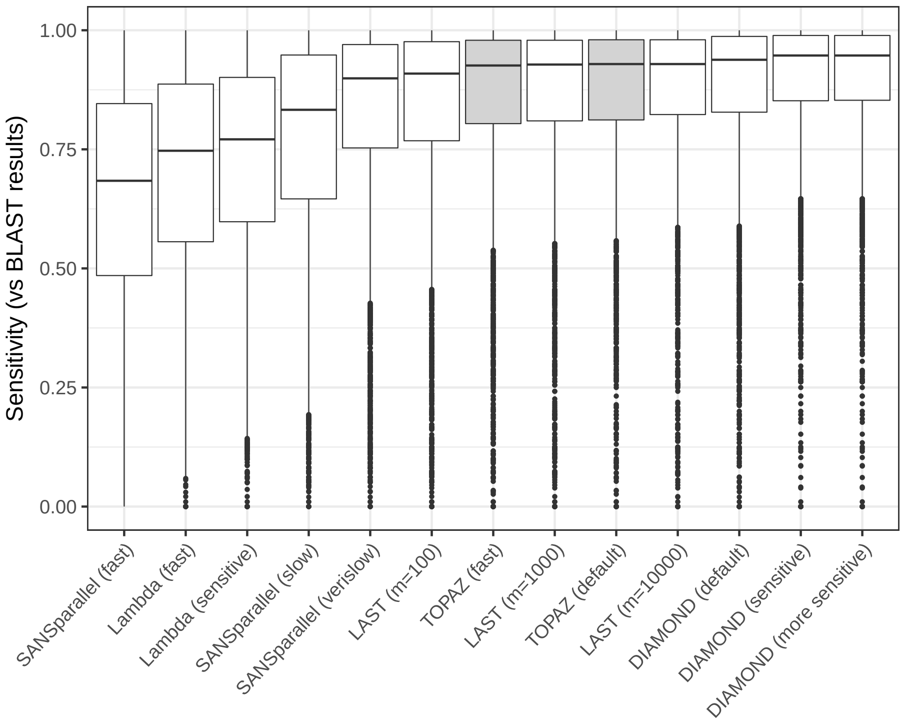

Figure S3: Distribution of sensitivity values per protein compared with BLAST results for each method. Methods are ordered by median sensitivity. TOPAZ modes are highlighted in grey. Number of hits = 100 and E-value threshold = 10^{-9}.

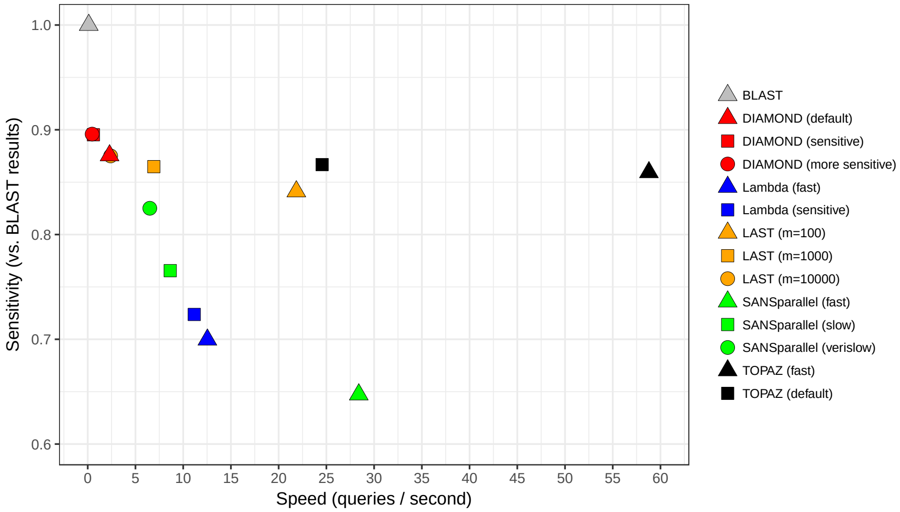

Figure S4: Speed versus average sensitivity across all proteins. The best speed was used for each method using up to 64 threads (all methods used 64 threads, with the exception of SANSparallel, which used 32). Number of hits = 100 and E-value threshold = 10^{-9}.
